# A genome-wide association study of energy intake and expenditure

**DOI:** 10.1371/journal.pone.0201555

**Published:** 2018-08-02

**Authors:** Lai Jiang, Kathryn L. Penney, Edward Giovannucci, Peter Kraft, Kathryn M. Wilson

**Affiliations:** 1 Program in Genetic Epidemiology and Statistical Genetics, Harvard T.H. Chan School of Public Health, Boston, Massachusetts, United States of America; 2 Department of Epidemiology, Harvard T.H. Chan School of Public Health, Boston, Massachusetts, United States of America; 3 Channing Division of Network Medicine, Department of Medicine, Brigham & Women’s Hospital/Harvard Medical School, Boston, Massachusetts, United States of America; 4 Department of Nutrition, Harvard T.H. Chan School of Public Health, Boston, Massachusetts, United States of America; Case Western Reserve University, UNITED STATES

## Abstract

Excessive energy intake or insufficient energy expenditure, which result in energy imbalance, contribute to the development of obesity. Obesity-related genes, such as *FTO*, are associated with energy traits. No genome-wide association studies (GWAS) have been conducted to detect the genetic associations with energy-related traits, including energy intake and energy expenditure, among European-ancestry populations. In this study, we conducted a genome-wide study using pooled GWAS including 12,030 European-ancestry women and 6,743 European-ancestry men to identify genetic variants associated with these two energy traits. We observed a statistically significant genome-wide SNP heritability for energy intake of 6.05% (95%CI = (1.76, 10.34), *P* = 0.006); the SNP heritability for expenditure was not statistically significantly greater than zero. We discovered three SNPs on chromosome 12q13 near gene *ANKRD33* that were genome-wide significantly associated with increased total energy intake among all men. We also identified signals on region 2q22 that were associated with energy expenditure among lean people. Body mass index related SNPs were found to be significantly associated with energy intake and expenditure through SNP set analyses. Larger GWAS studies of total energy traits are warranted to explore the genetic basis of energy intake, including possible differences between men and women, and the association between total energy intake and other downstream phenotypes, such as diabetes and chronic diseases.

## Introduction

Humans take in energy through protein, fat, and carbohydrate and expend energy in basal metabolism (reflecting by basic metabolic rate), thermogenesis, and physical activity [1, 2]. Energy traits, including energy balance, which is the difference between energy intake and energy expenditure, have been considered as key determinants of obesity [1, 3]. Body weight remains stable if energy intake equals energy expenditure; weight increases when energy intake exceeds energy expenditure [4]. According to National Health and Nutrition Examination Survey (NHANES), total daily energy intake increased in both men and women in recent years [5], which was consistent with the rapid increase in the prevalence of obesity among the US population [6, 7]. A recent large pathway analysis suggested that protein altering variants for body mass index (BMI) could control obesity through energy intake and expenditure biology [8]. Energy intake has also been associated with risk of chronic diseases, such as diabetes, colon cancer [9] and advanced or lethal prostate cancer [10].

There are conceptual and practical issues in considering energy intake and expenditure in epidemiologic studies. Conceptually, since energy intake and expenditure have multiple determinants (e.g., body size, metabolism, physical activity, over-consumption, menopausal status [11]), any association is difficult to interpret. Practically, there are considerable measurement errors from standard measures, i.e., food frequency questionnaires (FFQ) [12–14]. “Gold standard” measures, such as doubly labeled water (DLW), exist but are not widely used in large cohort studies due to the high cost and relatively complicated process. Seven-day food records have also been considered a “gold standard” of energy intake measurement, especially for validating other measures such as FFQs. However, food records place a high burden on study populations and the accuracy falls as the number of days increases [15]. Thus, most large cohorts use FFQs as a measurement of energy intake. Although FFQ measurements are noisy relative to "gold standard" measures, they are correlated with "gold standards" [16]. Studying FFQ measurements in a large sample is a practical option for studying the determinants and impact of energy consumption and expenditure, especially if the initial goal is to establish if there is any association between a factor and these traits.

The genetic architecture of energy traits is uncertain. Twin studies among European-ancestry populations suggested a familial aggregation of energy and macronutrient intake [17, 18] and that the correlations were higher in monozygotic (MZ) twins than in dizygotic (DZ) twins [19]; however, the familial effects are more likely to be attributed to shared environmental factors, such as the higher likelihood of eating together [19, 20]. The significant association between energy intake and *CLOCK* [21], a regulatory gene in the circadian system, as well as *FTO* [22, 23], an obesity-associated gene, were reported to increase total energy intake by candidate gene association studies. Nevertheless, the associations were inconsistent [24, 25]. A twin study showed that energy expenditure was impacted by genetic background in both MZ and DZ twins [26] and the familial effect on energy expenditure was confirmed in a segregation analysis [27]. A recent GWAS among American Indians reported variants on gene *GPR158* were significantly associated with energy expenditure; however, they suggested that such association cannot be replicated in other ethnic groups [28].

To the best of our knowledge, no genome-wide association study (GWAS) has been conducted to detect the genetic variation associated with total energy intake or energy expenditure among a European-ancestry population. In this study, we aimed to determine the genetic factors in the energy traits and to examine the association between energy traits and obesity from a genetic perspective.

## Materials and methods

### Study population

We used a pooled GWAS sample, including the Nurses’ Health Study (NHS) [29], Nurses’ Health Study II (NHS II) [30], and Health Professionals Follow-up Study (HPFS) [31]. NHS and NHS II were established in 1976 and 1989, respectively, aimed at studying women’s health. Women were followed every two years to update lifestyle and health information, with a validated semiquantitative FFQ every four years [32]. In NHS, blood samples were collected in 1989 and 2000, and cheek cells were collected in 2001. In NHS II, blood was collected in 1995, and check cells were collected in 2004. HPFS began in 1986, enrolling male health professionals (dentists and veterinarians, among others), with similar questionnaires and follow-up to that in NHS. Blood was collected in 1993 and cheek cells were collected in 2004. The study population for this analysis consists of participants with genotyping data from 11 nested case-control studies of various disease outcomes conducted in the three cohorts since 2007, who had also adequately completed at least two FFQs since 1986 for NHS and HPFS and 1991 for NHS II.

### Energy trait measures

The semiquantitative FFQ asked the participants to report their frequency of consumption for over 100 foods over the past year. A portion size for each food item was specified, and participants selected from nine frequency options ranging from “never or less than once per month” to “6+ per day”. *Daily energy intake* (kcals) for each food was computed by multiplying the frequency of consumption by the energy content of the specified portion from United States Department of Agriculture sources [33]. Participants with more than 70 missing food items on the FFQ, or extreme calculated daily energy intake (<600, >3500 for women, <800, >4200 for men) were excluded. Daily energy intake for each participant was calculated as the average of all available FFQs from baseline to 2010 (NHS, HPFS) or 2011 (NHS II) (mean 6.0 questionnaires, range 2–7). Averaging across FFQs was done to best represent diet over time and to reduce the impact of within-person measurement error [34].

*Daily energy expenditure* was calculated based on age and self-reported weight, height, and physical activity, according to the equations described in Gerrior *et al*. [2]. Age was used from the most recent questionnaire with a complete FFQ. Height was reported at baseline in each cohort, and weight was reported at baseline and every two years thereafter. Time per week spent in a list of leisure-time physical activity was reported every two years, and weekly expenditure of METs (metabolic equivalents) was calculated for each of these activities and for total activity. Weight and activity were averaged over all available questionnaires. Age at menopause was collected during the follow-up, and menopausal status was used to calculate pre- and postmenopausal energy intake and expenditure among women. We also calculated *daily energy balance* by subtracting daily energy expenditure from daily energy intake for additional analyses. Informed consents from study subjects were obtained. Study approval for each study was obtained from the Institutional Review Board (IRB) at Brigham and Women’s Hospital and the Harvard T.H. Chan School of Public Health.

### Genotyping and quality control

Since 2007, eleven case-control GWAS have been nested in the three cohorts with primary traits including breast cancer [35], pancreatic cancer [36], coronary heart disease [37], type 2 diabetes [38], kidney stone, advanced prostate cancer [39], glaucoma [40], gout, endometrial cancer [41], colon cancer [42], and mammographic density [43]. The genotyping and merging of each GWAS have been described in Lindstrom S *et al*.[44]. In total, the pooled GWAS dataset comprised 18,773 participants including 6,743 European-ancestry men from HPFS, 11,121 and 909 European-ancestry women from NHS and NHS II, respectively. Call rate, Hardy-Weinberg equilibrium, and other standard quality control filters were applied for samples and single nucleotide polymorphisms (SNPs) in each GWAS independently. We collapsed the eleven datasets into three combined GWAS datasets by platform family: the earlier generation of Illumina arrays (HumanHap), the Illumina OmniExpress array, and Affymetrix 6.0 array. Missing genotypes were imputed using Markov Chain (MACH) with 1000 Genomes Project ALL Phase I Integrated Release Version 3 Haplotypes as the reference panel. SNPs with a missing call rate > 5% or not originally genotyped in any platform in the merging process were excluded. SNPs with minor allele frequency (MAF) < 1% or imputation quality (r^2^) < 0.3 were excluded.

Pairwise identity by descent (IBD) was applied to identify duplicates and/or related individuals [44]. Individuals in pairs that were genotyped in more than one dataset were considered as expected duplicates. Pairs with a concordance rate > 0.999 but which were not expected duplicates were considered as unexpected duplicates. We removed 406 expected duplicates and 76 unexpected duplicates (i.e. 38 unexpected duplicate pairs) and flagged the pairs of related individuals (n = 13). Principal component analysis (PCA) was conducted by EIGENSTRAT [45] and outliers were checked with the top principal components.

### Statistical analysis

#### GWAS analysis

Logistic regression was applied for the GWAS analysis of the three pooled GWAS datasets with package ProbABEL [46]. We stratified the population by sex due to the potential differences in energy traits between women and men. We adjusted for three principal components accounting for subpopulation structure. Additionally, age, weight, height, and physical activity were adjusted in the energy intake analysis. SNP genotypes were coded as a dosage of the effect allele. X-linked SNPs were included. Fixed-effect meta-analysis across three family platforms was conducted to evaluate the SNP-level effect among men and women, respectively. We also implemented a fixed-effect meta-analysis across sex to test the overall SNP-level effect. Meta-analysis was conducted with software METAL [47] and Cochran’s Q statistic was used to test for heterogeneity between women and men. Additional analyses were performed by restricting the analysis population to overweight/obese (BMI≥25 kg/m^2^) and lean (BMI<25 kg/m^2^) subjects, respectively, due to concern about measurement error in energy intake and the potential underreporting of energy intake among overweight/obese population. Sensitivity analysis was conducted by applying deciles to rank energy intake as the phenotype among all subjects.

We estimated the genome-wide SNP heritability (*h*_*g*_^2^) using LD score regression [48], applied to the meta-analysis in the overall sample combining men and women.

#### SNP-set analyses

To determine the shared genetic contributions between energy traits and BMI, we implemented two SNP-set analyses: a weighted fixed-effects approach [49] and an unweighted random-effects (RE) model [50].

The fixed-effect approach tests for an association between a weighted genetic risk score for BMI and energy traits using summary genetic association statistics for each trait. Denoting the estimates of the effect of a BMI risk allele as *X* and the estimates of the effect of the same allele on an energy trait as *Y*, the inverse-variance weighted estimate of the average BMI risk allele effect on the energy trait is:

$${\hat{\beta }}_{IVW}=\frac{\sum_{k}X_{k}Y_{k}\sigma_{Yk}^{-2}}{\sum_{k}X_{k}^{2}\sigma_{Yk}^{-2}},\;se({\hat{\beta }}_{IVW})=\sqrt{\frac{1}{\sum_{k}x_{k}^{2}\sigma_{Yk}^{-2}}},$$ (1)

where $\sigma_{Yk}^{2}$ is the estimated variance *Y*.

The random-effects model assumes that each variant *k* belongs to one of two classes: an "effect" class and a "no effect" class. For variants in the "effect class," the effect size of each variant *k* is assumed to be drawn from a normal distribution with variance $\sigma_{Yk}^{2}$ and mean *μ*; variants in the "no effect" class are assumed to be drawn from a normal distribution with variance $\sigma_{Yk}^{2}$ and mean 0. A posterior probability *m*_*k*_ that variant *k* is in the "effect" class is calculated (assuming a N(0,0.2) prior on *μ*) and then used as a weight in a fixed-effects meta-analysis (i.e. replacing *X*_*k*_ with *m*_*k*_ in (1)). This approach has the advantage of not assuming that every BMI-associated SNP is associated with the tested energy trait; it also does not assume the effects of the SNPs that are associated with the energy trait are proportional to the SNPs' effect on BMI.

The summary statistics for genome-wide significant SNPs that were related to increasing BMI (*N*_*SNPs*_ = 76) in the SNP set were identified from the Genetic Investigation of ANthropometric Traits (GIANT) study [51].

## Results

Table 1 presents the descriptive statistics for the study population, stratified by sex. The average age at the most recent questionnaire was 74.6 y for women and 75.9 y for men. Of the 18,773 participants, more than half were overweight or obese (54.6% and 59.6% for women and men, respectively). The average physical activity intensity of women was half of men’s (17.0 and 33.4 MET-hr/week for women and men, respectively). Daily energy intake was 1724 and 2030 kcals among women and men, respectively; and daily energy expenditure was 1732 and 2348 kcals among women and men, respectively. The average reported energy intake was significantly lower than energy expenditure (*P* < 0.001) as has been previously reported [52]. The change in women’s average energy intake from premenopause to postmenopause was not significantly different (1812 and 1729 kcals for pre- and post-menopausal women, respectively; *P* = 0.88).

**Table 1:** Demographic characteristics of the study population (*N* = 18,773).

	Female	Male
	(*N* = 12,030)	(*N* = 6,743)
Average energy intake, kcals^a^	1724 (419)	2030 (507)
Average energy expenditure, kcals	1731 (216)	2348 (347)
Age at baseline, years	53.3 (7.5)	55.1 (8.7)
Age at most recent questionnaire, years	74.6 (8.2)	75.9 (8.0)
Height, inches	64.6 (2.4)	70.3 (2.6)
Weight^b^, pounds	157.4 (32.2)	184.3 (28.2)
Body mass index (BMI)^b^, kg/m^2^	26.5 (5.2)	26.2 (3.5)
BMI categories		
Normal (<25 kg/m^2^)	5461 (45.4)	2726 (40.4)
Overweight (≥ 25 kg/m^2^)	4017 (33.4)	3189 (47.3)
Obese (≥ 30 kg/m^2^)	2552 (21.2)	828 (12.3)
Physical activity^c^, MET-hours/week	17.0 (15.2)	33.4 (25.8)
Age at menopause, years^d^	49.2 (6.9)	-
Average premenopause energy intake, kcals^a^	1812 (502)	-
Average postmenopause energy intae, kcals^a^	1729 (430)	

*Note*: Continuous variables were displayed as mean (standard deviation). Categorical variables were displayed as number (proportion).

NHS = Nurses’ Health Study, NHS II = Nurses’ Health Study II, HPFS = Health Professionals Follow-up Study

^a^ Average from all available food frequency questionnaires. The number of questionnaire ranges from 2 to 7.

^b^ Average from all available questionnaires.

^c^ Data from 11,889 postmenopausal women during follow up.

^d^ Average from all women with available pre-menopause food frequency questionnaires (*N* = 3,651).

### GWAS analysis in all subjects

**Fig 1:**
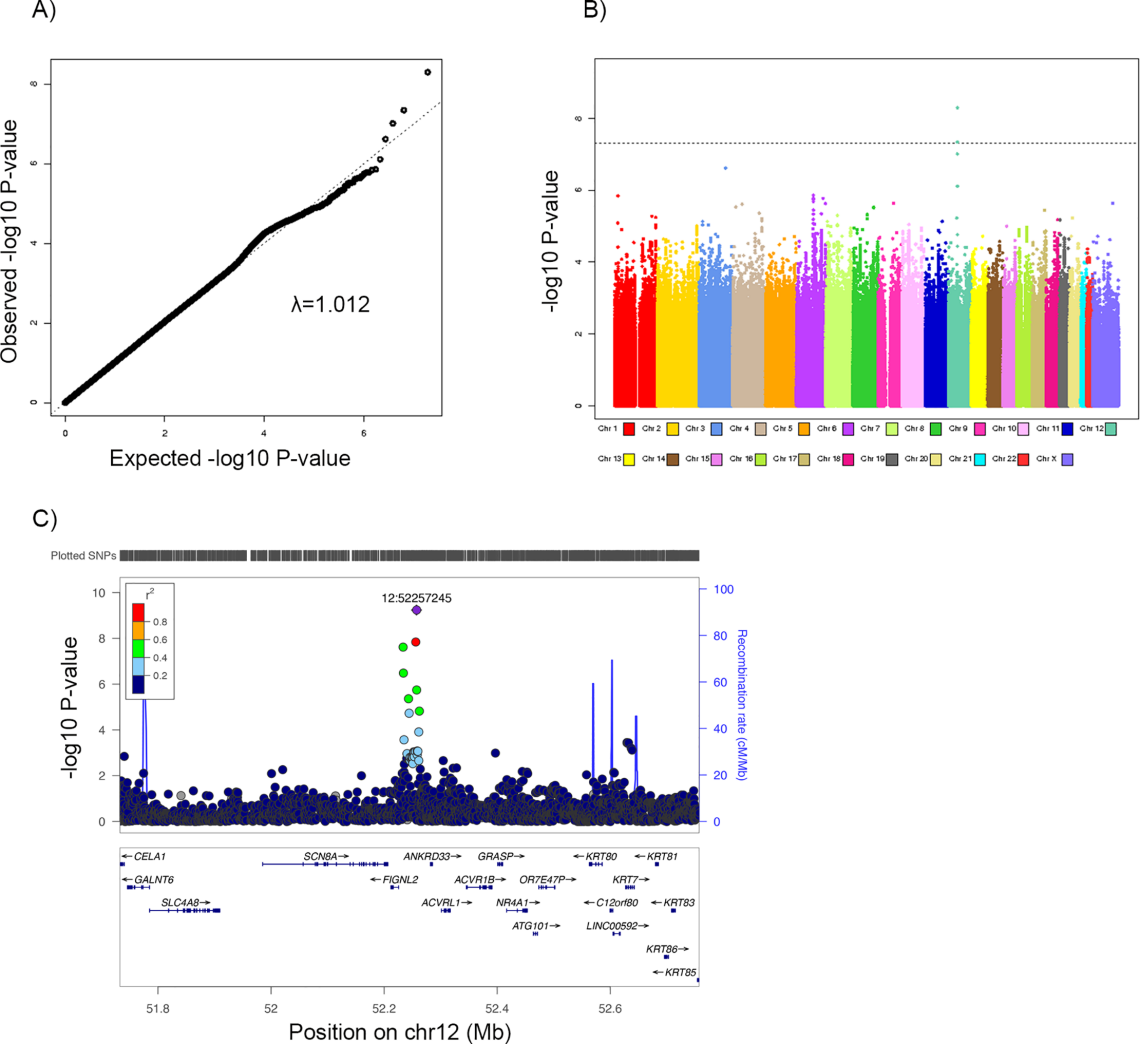
A) QQ plot for the SNP effect on daily energy intake for men. B) Manhattan plot for the SNP effect on daily energy intake for men. C) LocusZoom plot of the region associated with daily energy intake among men on chromosome 12q13.

**Fig 2:**
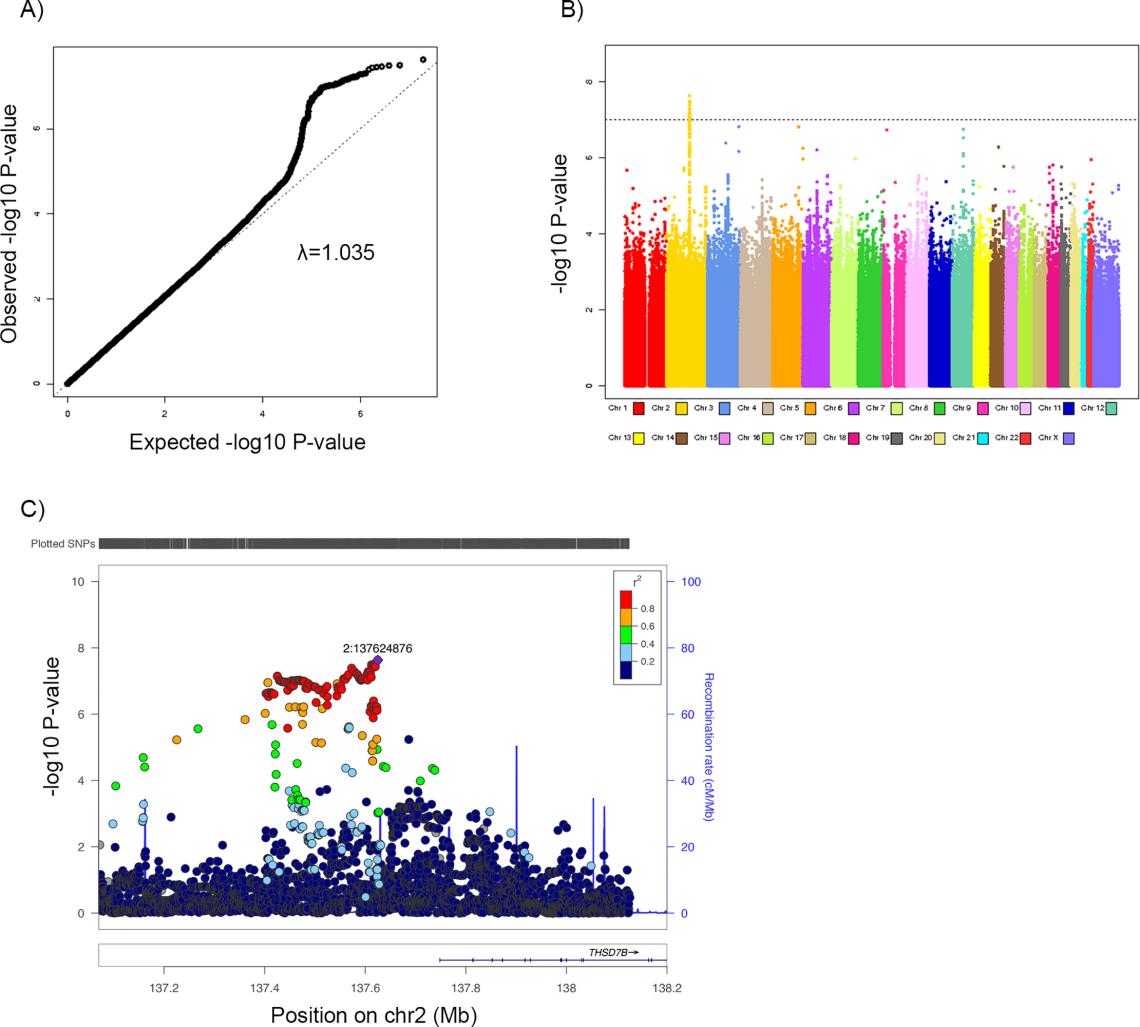
A) QQ plot for the SNP effect on daily energy expenditure for lean women and men. B) Manhattan plot for the SNP effect on daily energy expenditure for lean women and men. C) LocusZoom plot of the region associated with daily energy expenditure for lean women and men on chromosome 2q22.1.

Genome-wide significant (*P* < 5.0×10^−8^) variants were identified for daily energy traits among men (Table 2, Fig 1). rs10876214, an intronic SNP on chromosome 12q13 showed the strongest genome-wide significant association with a 58 kcal (95% CI = (39, 77), *P* = 9.86 × 10^−10^) increase in daily energy intake per effect allele among men. This SNP was also associated with a 56 kcal increase in daily energy balance per effect allele among men (95% CI = (36.6, 75.1), *P* = 1.22 × 10^−8^) (S2 Table). The SNP rs9669605 is in strong linkage disequilibrium (LD) (r^2^ > 0.8) with rs10876214 (Fig 2) and was also associated with energy intake: the effect allele of this SNP increased daily energy intake by 54 kcals(95% CI = (36, 73), *P* = 1.50 × 10^−8^). These SNPs are located near the *ANKRD33* (Ankyrin Repeat Domain 33) gene. Another signal at chromosome 12q13, rs10783478, mapped to approximately 7 kb downstream of *FLGNL2* (Fidgetin-like 2), was in modest LD (r^2^ = 0.59) with rs10876214 and increased energy intake by 54 kcal (95% CI = (35, 74), *P* = 3.0 × 10^−8^) per effect allele in men. No SNPs were genome-wide significant for energy intake among women. In the sensitivity analysis with deciles for the rank of energy intake, the p-values shrank for most signals as expected, considering the fact that the decile coding contains less information than the continuous coding. We observed genome-wide significance of rs9669605 and energy intake (*P* = 1.42 × 10^−8^) in the sensitivity analysis.

**Table 2:** Association between SNPs and daily energy traits among women, men, and meta-analyses combining women and men GWAS.

Marker^a^, alleles^b^, chromosome^c^, location^c^, and genes^d^		Subset	Total population (*N* = 18,774)			
			EAF	Effect (95% CI)	P_effect_ value	P_Het_ value^e^
**Daily energy intake**						
**rs10876214** (T, C)	Female		0.33	6 (-6, 17)	0.35	
12q13 (52257245)	Male		**0.32**	**58 (39, 77)**	**9.86 × 10**^**−10**^	
*ANKRD33*	Overall		0.32	22 (12, 3)	1.38 × 10^−5^	4.38 × 10^−6^
**rs9669605** (T, G)	Female		0.31	6 (-5, 18)	0.30	
12q13 (52254674)	Male		**0.30**	**54 (36, 73)**	**1.50 × 10**^**−8**^	
	Overall		0.30	21 (11, 30)	3.52 × 10^−5^	4.10 × 10^−5^
**rs10783478** (A, G)	Female		0.28	3 (-9, 17)	0.67	
12q13 (52232476)	Male		**0.27**	**54 (35, 74)**	**3.0 × 10**^**−8**^	
*FIGNL2*	Overall		0.27	18 (8, 28)	3.58 × 10^−4^	1.37 × 10^−5^
**Daily energy expenditure**						
**rs142343672** (A, G)	Female		**0.01**	**67 (45, 90)**	**5.16 × 10**^**−9**^	
11p15 (17871273)	Male		0.01	-5 (-69, 59)	0.89	
*LOC107984317*	Overall		**0.01**	**59 (38, 80)**	**4.85 × 10**^**−8**^	0.036
**rs146169233** (T, C)	Female		**0.01**	**52 (34, 70)**	**2.10 × 10**^**−8**^	
16p13 (9158320)	Male			Did not pass quality control		
	Overall			NA		
**rs61957289** (C, T)	Female		**0.99**	**53 (35, 72)**	**1.27 × 10**^**−8**^	
13q22 (74400573)	Male		0.98	-15 (-69, 39)	0.58	
*KLF12*	Overall		0.98	46 (29, 63)	1.90 × 10^−7^	0.019

*Note*: Results from the unconditional logistic regression of the genotypes in the pooled GWAS for total subjects (12,031 women and 6,743 men. The analyses were adjusted for five principal components accounting for population substructure. Additionally, age, height, weight, and physical activity were adjusted for in energy intake.

EAF, effect allele frequency; CI, confidence interval; Het, heterogeneity.

^a^NCBI dbSNP identifier

^b^effect allele, reference allele

^c^chromosome and NCBI Human Genome Build 37 location

^d^closest genes, genes located within 25 kb

^e^Heterogeneity between women and men

We detected three SNPs that were genome-wide associated with daily energy expenditure (Table 2). Locus rs142343672 at chromosome 11p15 increased expenditure in women by 67 kcal (95% CI = (45, 90), *P* = 5.16 × 10^−9^) and in women and men combined by 59 kcal (95% CI = (38, 80), *P* = 4.85 × 10^−8^). Another two loci, rs146169233 and rs61957289, increased expenditure in women by 52 kcal (95% CI = (34, 70), *P* = 2.10 × 10^−8^) and 53 kcal (95% CI = (35, 72), *P* = 1.27 × 10^−8^) per effect allele, respectively.

Aside from these individual SNP associations, we also observed a collective contribution of common genetic variants to energy intake and expenditure. The estimate of genome-wide SNP heritability for energy intake and energy expenditure was h_g_^2^ = 6.05% (95%CI = (1.76, 10.34), *P* = 0.006) and h_g_^2^ = -2.96% (95% CI = (-7.33, 1.41), *P* = 0.184), respectively. (The LD score estimator of h_g_^2^ is unbiased but not constrained to the interval [0,1].)

### GWAS analysis in overweight/obese and lean subjects

Table 3 presents the association between SNPs and daily energy traits among overweight/obese women and men. We detected locus rs111431452, which mapped to gene *ADORA3* (Adenosine A3 Receptors), significantly increased daily energy intake in this population by 145 kcal (95% CI = (94, 197), *P* = 3.59 × 10^−8^) per effect allele. The effect of rs111431452 on energy intake was consistent between men and women (*P*_Het_ *=* 0.29) but was not genome-wide significant when stratifying the population by sex.

**Table 3:** Association between SNPs and daily energy traits among overweight and obese (BMI ≥ 25 kg/m^2^) women, men, and meta-analyses combining women and men.

Marker^a^, alleles^b^, chromosome^c^, location^c^, and genes^d^		Subset	Overweight/Obese population (*N* = 10,583)			
			EAF	Effect (95% CI)	P_effect_ value	P_Het_ value^e^
**Daily energy intake**						
**rs111431452** (A, T)	Female		0.02	128 (67, 189)	4.19 × 10^−5^	
1p31 (112056878)	Male		0.02	189 (93, 286)	1.27 × 10^−4^	
*ADORA3*	Overall		**0.02**	**145 (94, 197)**	**3.59 × 10**^**−8**^	0.29
**Daily energy expenditure**						
**rs62131523** (G, A)	Female		0.96	-14 (-36, 8)	0.21	
2p24 (17746338)	Male		**0.96**	**124 (80, 169)**	**3.41 × 10**^**−8**^	
*VSNL1*	Overall		0.96	13 (-6, 33)	0.19	3.71 × 10^−8^
**rs7162556** (A, G)	Female		0.10	2 (-11, 16)	0.73	
15q25 (80675244)	Male		**0.10**	**-72 (-97, -46)**	**3.66 × 10**^**−8**^	
	Overall		0.10	-13 (-25, -2)	0.03	4.45 × 10^−7^

*Note*: Results from the unconditional logistic regression of the genotypes in the pooled GWAS for overweight/obese subjects only (6,563 women and 4,020 men). The analyses were adjusted for five principal components accounting for population substructure. Additionally, age, height, weight, and physical activity were adjusted for in energy intake.

EAF, effect allele frequency; CI, confidence interval; Het, heterogeneity.

^a^NCBI dbSNP identifier

^b^effect allele, reference allele

^c^chromosome and NCBI Human Genome Build 37 location

^d^closest genes, genes located within 25 kb

^e^Heterogeneity between women and men

Two SNPs were significantly associated with energy expenditure among overweight/obese men (Table 3). At chromosome 2p24, SNP rs62131523 is an intron variant of gene VSNL1 (visinin-like 1). We found the effect allele of rs62131523 significantly increased energy expenditure among overweight and obese men by 124 kcal (95% CI = (80, 169), *P =* 3.41 × 10^−8^). SNP rs7162556 at chromosome 15q25 decreased energy expenditure of men with high BMI (effect = -72 kcal per effect allele, 95% CI = (-97, -46), and *P* = 3.66 × 10^−8^). Both of the two SNPs showed significant sex-differential effects on energy expenditure in the overweight and obese population (*P*_Het_ *=* 3.71 × 10^−8^ for rs62131523 and *P*_Het_ *=* 4.45 × 10^−7^ for rs7162556). In the analysis of daily energy balance, the minor allele of rs2723689 significantly decreased energy balance by 78 kcal among overweight/obese men (95% CI = (-107, -50), *P* = 8.02 × 10^−8^) (S1 Table). The effect of rs2723689 on energy balance was significantly different between women and men (*P*_Het_ = 4.44 × 10^−5^).

Table 4 shows the association between SNPs and daily energy traits among lean women and men. We identified a region on chromosome 2q22 with eight SNPs which are in strong LD (r^2^ > 0.8) that were significantly associated with daily energy expenditure among lean women and men (Fig 2). SNP rs55691047 had the strongest association with expenditure (effect = 21 kcal per copy of the risk allele, 95% CI = (14, 29), *P* = 2.35 × 10^−8^). SNP rs7138102 on chromosome 12q14 was associated with energy expenditure among lean women (effect = -18 kcal per copy of the risk allele, 95% CI = (-25, -12), and *P* = 3.68 × 10^−8^) but not among lean men.

**Table 4:** Association between SNPs and daily energy traits among lean (BMI < 25kg/m^2^) women, men, and meta-analyses combining women and men.

Marker^a^, alleles^b^, chromosome^c^, location^c^, and genes^d^	Subset		Lean population (*N* = 8,187)				
			EAF		Effect (95% CI)	P_effect_ value	P_Het_ value^e^
**Daily energy expenditure**							
**rs7138102** (A, G)		Female	**0.40**	**-18 (-25, -12)**		**3.68 × 10**^**−8**^	
12q14 (66353891)		Male	0.42	-3 (-19, 13)		0.73610	
		Overall	0.41	-16 (-22, -10)		1.80 × 10^−7^	0.07
**rs13002862** (A, C)		Female	0.21	19 (11, 27)		2.52 × 10^−6^	
2q22 (137613935)		Male	0.19	32 (12, 52)		0.00198	
		Overall	**0.21**	**21 (13, 28)**		**3.55 × 10**^**−8**^	0.25
**rs35893283** (T, C)		Female	0.21	19 (11, 27)		2.54 × 10^−6^	
2q22 (137618545)		Male	0.19	32 (12, 52)		0.00203	
		Overall	**0.21**	**21 (13, 28)**		**3.63 × 10**^**−8**^	0.25
**rs72844022** (G, A)		Female	0.21	19 (11, 27)		2.32 × 10^−6^	
2q22 (137610788)		Male	0.19	32 (11, 52)		0.00198	
		Overall	**0.21**	**21 (13, 28)**		**3.25 × 10**^**−8**^	0.25
**rs6720647** (G, A)		Female	0.20	20 (12, 28)		1.52 × 10^−6^	
2q22 (137615688)		Male	0.18	31 (10, 52)		0.00390	
		Overall	**0.20**	**21 (14, 29)**		**3.19 × 10**^**−8**^	0.35
**rs55691047** (G, A)		Female	0.21	19 (11, 27)		1.80 × 10^−6^	
2q22 (137624876)		Male	0.19	32 (12, 53)		0.00180	
		Overall	**0.21**	**21 (14, 29)**		**2.35 × 10**^**−8**^	0.24
**rs34197312** (G, C)		Female	0.21	19 (11, 27)		3.84 × 10^−6^	
2q22 (137608941)		Male	0.19	33 (12, 53)		0.00162	
		Overall	**0.21**	**21 (13, 28)**		**4.93 × 10**^**−8**^	0.21
**rs35845238** (G, C)		Female	0.21	19 (11, 27)		2.39 × 10^−6^	
2q22 (137617708)		Male	0.19	32 (12, 52)		0.00208	
		Overall	**0.21**	**21 (13, 28)**		**3.45 × 10**^**−8**^	0.25
**rs34399632** (G, A)		Female	0.21	19 (11, 27)		2.39 × 10^−6^	
2q22 (137571174)		Male	0.19	31 (11, 51)		0.00270	
		Overall	**0.21**	**21 (13, 28)**		**4.07 × 10**^**−8**^	0.29

*Note*: Results from the unconditional logistic regression of the genotypes in the pooled GWAS for lean subjects (5,461 women and 2,726 men). The analyses were adjusted for five principal components accounting for population substructure. Additionally, age, height, weight, and physical activity were adjusted for in energy intake.

EAF, effect allele frequency; CI, confidence interval; Het, heterogeneity.

^a^NCBI dbSNP identifier

^b^effect allele, reference allele

^c^chromosome and NCBI Human Genome Build 37 location

^d^closest genes, genes located within 25 kb

^e^Heterogeneity between women and men

### SNP set analysis

**Fig 3:**
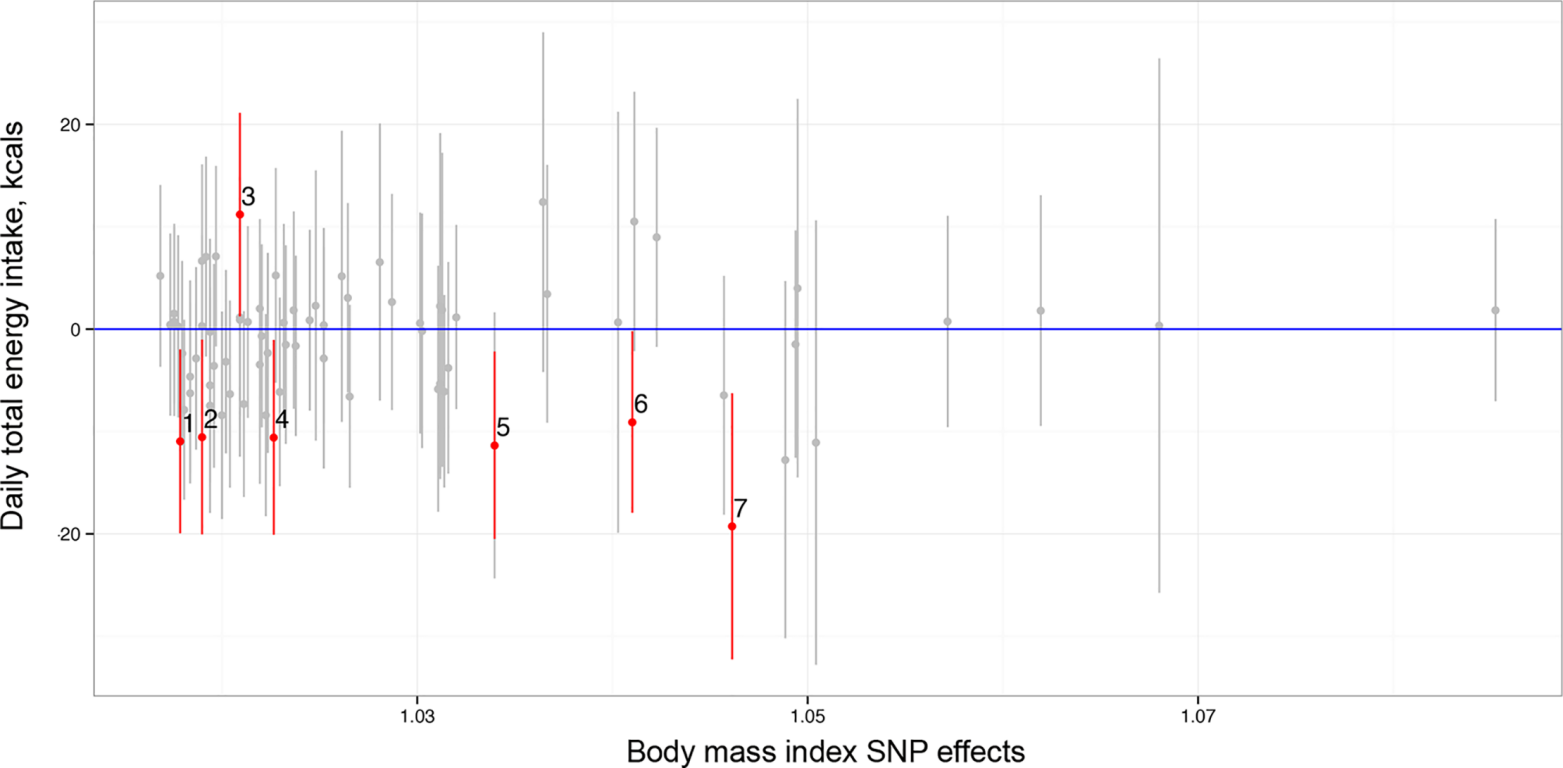
Plot of the regression coefficients for the effect of BMI-increasing alleles on energy intake (and their 95% confidence intervals) as function of per-allele effect on BMI. The marked SNPs in panel (a) are: 1, rs11583200 (Chr 1:50559820); 2, rs9400239 (Chr 6:108977663); 3, rs11126666 (Chr 2:26928811); 4, rs17405819 (Chr 8:76806584); 5, rs3101336 (Chr 1:72751185); 6, rs10938397 (Chr 4:45182527); 7, rs1516725 (Chr3:185824004).

In the SNP set analysis, the fixed effect analysis suggested that the alleles that increase BMI were associated with a decrease in daily energy intake (*P*_Fixed_ = 0.015) (Fig 3) among the study population combining women and men. The random effects analysis supported the finding (*P*_Random_ = 0.008) and suggested that alleles associated with BMI were also associated with expenditure without considering the direction of the alleles’ effect on expenditure (*P*_Random_ = 0.016).

## Discussion

To the best of our knowledge, this is the first reported GWAS pertaining to total daily energy intake and energy expenditure among a European-ancestry population. We established that energy intake is a heritable trait, with h_g_^2^ = 6.05% (95%CI = (1.76, 10.34)). Although small, the estimated h_g_^2^ for energy intake is statistically significant (*P* = 0.006). The modest h_g_^2^ value likely reflects the fact that energy intake is a complex, challenging-to-measure trait.

We identified a region on 12q13 that was associated with total energy intake among men and a region on 2q22 that was associated with total energy expenditure. We also found a shared genetic contribution between increasing BMI and decreasing energy intake among women and men.

### Newly discovered signals associated with energy traits

At chromosome 12q13, the SNP showing the strongest association with energy intake was rs10876214. This SNP is located approximately 25 kb upstream of *ANKRD33* (encoding an ankrytin repeat-containing protein). Another SNP associated with energy intake at this locus, rs107834787, is located 7 kb downstream of *FIGNL2* (fidgetin-like 2). Previous *in silico*, *in vitro* and *in vivo* experiments have linked these genes to photoreceptor signaling (*ANKRD33*) and ATP binding (*FIGNL2*) [53–55], but the mechanisms linking genetic variation in this region to energy intake are unclear and require further study.

Among lean men and women, we identified eight SNPs at region 2q22.1 that were associated with energy expenditure. These SNPs are in modest LD (0.4<r^2^<0.6) with SNPs that have been previously reported to be associated with adiposity-related traits, including body weight [56], waist:hip ratio [57], and cardiovascular functions [58]. These previously-reported SNPs are in an intron of *THSD7B* (thrombospondin type 1 domain containing 7B), which expresses most tissue-specific proteomes in fat tissues [59].

### Replication of previously identified energy trait-related genes

*FTO* (alpha-ketoglutarate dependent dioxygenase) has been identified as an obesity-associated gene [60]. Previous studies reported inconsistent effects of *FTO* on both energy intake and energy expenditure [22–25, 61]. In our study, no *FTO* genetic effect on energy intake or energy expenditure was detected. None of another three previously reported energy trait-related genes, including *CLOCK* [21], *MC4R* [62], and *FGF21* [63], showed significant effects in our GWAS nor are in LD with our top SNPs. The discrepancy between the effect of these genes on energy traits may due to the different food items that investigators used to calculate energy intake [23], the different methods to measure energy expenditure, or differences between study populations [22].

### Association between obesity and energy traits

We observed that the established SNPs for increasing BMI decreased energy intake. The inverse association may due to the fact that obese people are less likely to feel hungry than lean people [64]. Such difference may be caused by chemicals in human subjects, such as the glucagon-like peptide-1 (7–36) amide (GLP-1) which could suppress energy intake [65] and feelings of hunger [66] among obese people. Neuroimaging also demostrated different brain responses to meals between obese and non-obese groups [67]. Obesity could also alter the gene expression of bacterial genes and metabolic pathways in gut microbiome among obese people [68, 69], which triggers feeding behaviors in human subjects [70]. Alternatively, obese individuals may be more likely to underreport energy intake on dietary questionnaires [71, 72].

### Limitations and strengths

Our study is subject to several limitations. The major concern is measurement error in energy intake assessed via FFQ. Correlations between energy intake from a single FFQ with two to four weeks of diet records are in the range of 0.3 to 0.4 [73]. We used the average of between two and seven FFQs in order to somewhat reduce within-person measurement error [34]; however, our assessment is still far from perfect. The measurement error will be unrelated to genetic factors, so our results should be unbiased, but our statistical power is greatly attenuated. The large sample size of our analysis compensates for the attenuated power and enables us to at least discover the large effects. This perspective was justified by the sensitivity analysis in which we used deciles for the rank of energy intake as phenotype and re-ran the analysis among all subjects. Second, in the SNP set analysis, we only measured the effect of BMI-related allele sets on energy traits, but we could not comprehensively or reliably test for the association between energy intake and expenditure alleles and BMI because our discovery GWAS had limited power. Larger GWAS studies of total energy traits are warranted to identify more genetic risk alleles and the newly identified signals from our study can be candidates for the future studies. Moreover, a potential future study is to see if energy intake in smaller samples with more accurate measures of energy intake could replicate the effects of our identified SNPs. Previous studies suggested that energy density may modify the relationship between obesity and energy intake [74] as well as obesity and energy expenditure [75]. Future studies may explore the genetic variations that contribute to energy density and the possible shared genetic components between energy density and obesity to understand the underlying mechanisms of obesity and the energy system.

To the best of our knowledge, there are no previous GWAS that explored the genetic variation in energy traits among European-ancestry populations. The strengths of our study include: the comprehensive and valid genetic information in our combined GWAS subjects [44] and repeated assessment of dietary intakes via a validated semiquantitative food frequency questionnaire [32]. Another strength of our study is that we used results from a previous large GWAS of BMI to identify candidate sets of SNPs to include in allele-score and SNP-set analyses. This allowed us to explore the genetic contribution to BMI that is shared with energy intake and energy expenditure.

## Conclusions

In summary, we demonstrated that energy intake is a heritable trait and that three SNPs at 12q13 were associated with total daily energy intake among European-ancestry men. Eight SNPs at 2q22.1 with high LD were associated with energy expenditure among lean European-ancestry women and men. Our findings suggest there is a shared genetic contribution to BMI and energy intake as well as BMI and energy expenditure. Further investigation is warranted to replicate our findings and detect the additional genetic variants associated with energy intake and expenditure.

## Supporting information

S1 Fig QQ plot for the SNP effect on A) daily energy intake; B) daily energy expenditure among women and men (left), women (middle), and men (right).
(TIF)

S2 Fig Manhattan plot for the SNP effect on A) daily energy intake; B) daily energy expenditure among women and men (left), women (middle), and men (right).
(TIF)

S3 Fig QQ plot for the SNP effect on A) daily energy intake; B) daily energy expenditure among overweight and obese women and men (left), women (middle), and men (right).
(TIF)

S4 Fig Manhattan plot for the SNP effect on A) daily energy intake; B) daily energy expenditure among overweight and obese women and men (left), women (middle), and men (right).
(TIF)

S5 Fig QQ plot for the SNP effect on A) daily energy intake; B) daily energy expenditure among lean women and men (left), women (middle), and men (right).
(TIF)

S6 Fig Manhattan plot for the SNP effect on A) daily energy intake; B) daily energy expenditure among lean women and men (left), women (middle), and men (right).
(TIF)

S1 Table Association between SNPs and daily energy balance among women, men, and meta-analyses combining women and men in the pooled GWAS. (PDF)

S2 Table Association between top SNPs that were identified from the total population and daily energy traits among overweight/obese and lean women, men, and meta-analyses combining women and men in the pooled GWAS. (PDF)

S3 Table Information of ßs and standard errors of SNP-set analysis for the association between body mass index and energy traits in total population. (PDF)

## References

[1] Spiegelman BM, Flier JS. Obesity and the regulation of energy balance. Cell. 2001;104(4):531–43. 11239410 10.1016/s0092-8674(01)00240-9

[2] Gerrior S, Juan W, Peter B. An easy approach to calculating estimated energy requirements. Preventing chronic disease. 2006;3(4). PMC1784117 16978504

[3] Nishida C, Uauy R, Kumanyika S, Shetty P. The joint WHO/FAO expert consultation on diet, nutrition and the prevention of chronic diseases: process, product and policy implications. Public health nutrition. 2004;7(1a):245–50. 14972063 10.1079/phn2003592

[4] Hill JO, Wyatt HR, Peters JC. Energy balance and obesity. Circulation. 2012;126(1):126–32. 10.1161/CIRCULATIONAHA.111.087213 22753534 PMC3401553

[5] Control CfD Prevention. Trends in intake of energy and macronutrients—United States, 1971–2000. MMWR Morbidity and mortality weekly report. 2004;53(4):80 14762332

[6] Flegal KM, Carroll MD, Kuczmarski RJ, Johnson CL. Overweight and obesity in the United States: prevalence and trends, 1960–1994. International journal of obesity. 1998;22(1):39–47. 9481598 10.1038/sj.ijo.0800541

[7] Weight W. Overweight and Obesity Statistics.

[8] Turcot V, Lu Y, Highland HM, Schurmann C, Justice AE, Fine RS, et al Protein-altering variants associated with body mass index implicate pathways that control energy intake and expenditure in obesity. Nature genetics. 2018;50(1):26 10.1038/s41588-017-0011-x 29273807 PMC5945951

[9] Lyon JL, Mahoney AW, West DW, Gardner JW, Smith KR, Sorenson AW, et al Energy intake: its relationship to colon cancer risk. Journal of the National Cancer Institute. 1987;78(5):853–61. 3033383

[10] Platz EA, Leitzmann MF, Michaud DS, Willett WC, Giovannucci E. Interrelation of energy intake, body size, and physical activity with prostate cancer in a large prospective cohort study. Cancer research. 2003;63(23):8542–8. 14679023

[11] Poehlman ET, Toth MJ, Gardner AW. Article RETRACTED: Changes in energy balance and body composition at menopause: A controlled longitudinal study. Annals of internal medicine. 1995;123(9):673–5. 7574222 10.7326/0003-4819-123-9-199511010-00005

[12] Willett W, Stampfer MJ. Total energy intake: implications for epidemiologic analyses. American journal of epidemiology. 1986;124(1):17–27. 3521261 10.1093/oxfordjournals.aje.a114366

[13] Willett WC, Howe GR, Kushi LH. Adjustment for total energy intake in epidemiologic studies. The American journal of clinical nutrition. 1997;65(4):1220S–8S. 9094926 10.1093/ajcn/65.4.1220S

[14] Hu FB, Stampfer MJ, Rimm E, Ascherio A, Rosner BA, Spiegelman D, et al Dietary fat and coronary heart disease: a comparison of approaches for adjusting for total energy intake and modeling repeated dietary measurements. American journal of epidemiology. 1999;149(6):531–40. 10084242 10.1093/oxfordjournals.aje.a009849

[15] Johnson RK. Dietary intake—how do we measure what people are really eating? Obesity. 2002;10(S11). 10.1038/oby.2002.192 12446861

[16] Siebelink E, Geelen A, de Vries JH. Self-reported energy intake by FFQ compared with actual energy intake to maintain body weight in 516 adults. British journal of nutrition. 2011;106(2):274–81. 10.1017/S0007114511000067 21338536

[17] Faith MS, Keller KL, Johnson SL, Pietrobelli A, Matz PE, Must S, et al Familial aggregation of energy intake in children. The American journal of clinical nutrition. 2004;79(5):844–50. 10.1093/ajcn/79.5.844 15113724

[18] Hasselbalch AL, Heitmann BL, Kyvik KO, Sørensen TI. Studies of twins indicate that genetics influence dietary intake. The Journal of nutrition. 2008;138(12):2406–12. 10.3945/jn.108.087668 19022965

[19] Heller R, O'Connell D, Roberts D, Allen J, Knapp J, Steele P, et al Lifestyle factors in monozygotic and dizygotic twins. Genetic Epidemiology. 1988;5(5):311–21. 10.1002/gepi.1370050503 3215506

[20] Perusse L, Tremblay A, Leblanc C, Cloninger C, Reich T, Rice J, et al Familial resemblance in energy intake: contribution of genetic and environmental factors. The American journal of clinical nutrition. 1988;47(4):629–35. 10.1093/ajcn/47.4.629 3354487

[21] Garaulet M, Lee Y-C, Shen J, Parnell LD, Arnett DK, Tsai MY, et al Genetic variants in human CLOCK associate with total energy intake and cytokine sleep factors in overweight subjects (GOLDN population). European Journal of Human Genetics. 2010;18(3):364–9. 10.1038/ejhg.2009.176 19888304 PMC2987209

[22] Cecil JE, Tavendale R, Watt P, Hetherington MM, Palmer CN. An obesity-associated FTO gene variant and increased energy intake in children. New England Journal of Medicine. 2008;359(24):2558–66. 10.1056/NEJMoa0803839 19073975

[23] Haupt A, Thamer C, Staiger H, Tschritter O, Kirchhoff K, Machicao F, et al Variation in the FTO gene influences food intake but not energy expenditure. Experimental and Clinical Endocrinology & Diabetes. 2009;117(04):194–7. 19053021 10.1055/s-0028-1087176

[24] Liu G, Zhu H, Lagou V, Gutin B, Stallmann-Jorgensen IS, Treiber FA, et al FTO variant rs9939609 is associated with body mass index and waist circumference, but not with energy intake or physical activity in European-and African-American youth. BMC medical genetics. 2010;11(1):1. 20377915 10.1186/1471-2350-11-57 PMC2864242

[25] Hakanen M, Raitakari OT, Lehtimä T, Peltonen N, Pahkala K, Sillanmaki L, et al FTO genotype is associated with body mass index after the age of seven years but not with energy intake or leisure-time physical activity. The Journal of Clinical Endocrinology & Metabolism. 2009;94(4):1281–7. 19158205 10.1210/jc.2008-1199

[26] Fontaine E, Savard R, Tremblay A, Despres J, Poehlman E, Bouchard C. Resting metabolic rate in monozygotic and dizygotic twins. Acta geneticae medicae et gemellologiae: twin research. 1985;34(1–2):41–7. 4050294 10.1017/s0001566000004906

[27] Ravussin E, Bogardus C. Relationship of genetics, age, and physical fitness to daily energy expenditure and fuel utilization. The American Journal of Clinical Nutrition. 1989;49(5):968–75. 2655422 10.1093/ajcn/49.5.968

[28] Piaggi P, Masindova I, Muller YL, Mercader J, Wiessner GB, Chen P, et al A genome-wide association study using a custom genotyping array identifies variants in GPR158 associated with reduced energy expenditure in American Indians. Diabetes. 2017;66(8):2284–95. 10.2337/db16-1565 28476931 PMC5521859

[29] Colditz GA, Hankinson SE. The Nurses' Health Study: lifestyle and health among women. Nature Reviews Cancer. 2005;5(5):388–96. 10.1038/nrc1608 15864280

[30] Tworoger SS, Sluss P, Hankinson SE. Association between plasma prolactin concentrations and risk of breast cancer among predominately premenopausal women. Cancer Research. 2006;66(4):2476–82. 10.1158/0008-5472.CAN-05-3369 16489055

[31] Giovannucci E, Pollak M, Liu Y, Platz EA, Majeed N, Rimm EB, et al Nutritional predictors of insulin-like growth factor I and their relationships to cancer in men. Cancer Epidemiology Biomarkers & Prevention. 2003;12(2):84–9. 12582016

[32] Willett WC, Sampson L, Stampfer MJ, Rosner B, Bain C, Witschi J, et al Reproducibility and validity of a semiquantitative food frequency questionnaire. American journal of epidemiology. 1985;122(1):51–65. 4014201 10.1093/oxfordjournals.aje.a114086

[33] USDA. Composition of Foods—Raw, Processed, and Prepared, 1963–1988.: U.S. Government Printing Office Washington, DC; 1989.

[34] Willett W. Nutritional epidemiology: Oxford University Press; 2012 10.1097/EDE.0b013e31825afb0b

[35] Hunter DJ, Kraft P, Jacobs KB, Cox DG, Yeager M, Hankinson SE, et al A genome-wide association study identifies alleles in FGFR2 associated with risk of sporadic postmenopausal breast cancer. Nature genetics. 2007;39(7):870–4. 10.1038/ng2075 17529973 PMC3493132

[36] Amundadottir L, Kraft P, Stolzenberg-Solomon RZ, Fuchs CS, Petersen GM, Arslan AA, et al Genome-wide association study identifies variants in the ABO locus associated with susceptibility to pancreatic cancer. Nature genetics. 2009;41(9):986–90. 10.1038/ng.429 19648918 PMC2839871

[37] Jensen MK, Pers TH, Dworzynski P, Girman CJ, Brunak S, Rimm EB. Protein interaction-based genome-wide analysis of incident coronary heart disease. Circulation: Cardiovascular Genetics. 2011;4(5):549–56. 10.1161/CIRCGENETICS.111.960393 21880673 PMC3197770

[38] Qi L, Cornelis MC, Kraft P, Stanya KJ, Kao WL, Pankow JS, et al Genetic variants at 2q24 are associated with susceptibility to type 2 diabetes. Human molecular genetics. 2010;19(13):2706–15. 10.1093/hmg/ddq156 20418489 PMC2883345

[39] Schumacher FR, Berndt SI, Siddiq A, Jacobs KB, Wang Z, Lindstrom S, et al Genome-wide association study identifies new prostate cancer susceptibility loci. Human molecular genetics. 2011;20(19):3867–75. 10.1093/hmg/ddr295 21743057 PMC3168287

[40] Wiggs JL, Kang JH, Yaspan BL, Mirel DB, Laurie C, Crenshaw A, et al Common variants near CAV1 and CAV2 are associated with primary open-angle glaucoma in caucasians from the United States. Human molecular genetics. 2011:ddr382. 10.1093/hmg/ddr382 PMC3209825 21873608

[41] De Vivo I, Prescott J, Setiawan VW, Olson SH, Wentzensen N, Attia J, et al Genome-wide association study of endometrial cancer in E2C2. Human genetics. 2014;133(2):211–24. 10.1007/s00439-013-1369-1 24096698 PMC3898362

[42] Peters U, Jiao S, Schumacher FR, Hutter CM, Aragaki AK, Baron JA, et al Identification of genetic susceptibility loci for colorectal tumors in a genome-wide meta-analysis. Gastroenterology. 2013;144(4):799–807. e24. 10.1053/j.gastro.2012.12.020 23266556 PMC3636812

[43] Stevens KN, Lindstrom S, Scott CG, Thompson D, Sellers TA, Wang X, et al Identification of a novel percent mammographic density locus at 12q24. Human molecular genetics. 2012;21(14):3299–305. 10.1093/hmg/dds158 22532574 PMC3384385

[44] Lindstrom S, Loomis S, Turman C, Huang H, Huang J, Aschard H, et al A comprehensive survey of genetic variation in 20,691 subjects from four large cohorts. bioRxiv. 2016:083030. 10.1371/journal.pone.0173997 PMC5354293 28301549

[45] Price AL, Patterson NJ, Plenge RM, Weinblatt ME, Shadick NA, Reich D. Principal components analysis corrects for stratification in genome-wide association studies. Nature genetics. 2006;38(8):904–9. 10.1038/ng1847 16862161

[46] Aulchenko YS, Struchalin MV, van Duijn CM. ProbABEL package for genome-wide association analysis of imputed data. BMC bioinformatics. 2010;11(1):1. 20233392 10.1186/1471-2105-11-134 PMC2846909

[47] Willer CJ, Li Y, Abecasis GR. METAL: fast and efficient meta-analysis of genomewide association scans. Bioinformatics. 2010;26(17):2190–1. 10.1093/bioinformatics/btq340 20616382 PMC2922887

[48] Bulik-Sullivan BK, Loh P-R, Finucane HK, Ripke S, Yang J, Patterson N, et al LD Score regression distinguishes confounding from polygenicity in genome-wide association studies. Nature genetics. 2015;47(3):291 10.1038/ng.3211 25642630 PMC4495769

[49] Burgess S, Butterworth A, Thompson SG. Mendelian randomization analysis with multiple genetic variants using summarized data. Genetic epidemiology. 2013;37(7):658–65. 10.1002/gepi.21758 24114802 PMC4377079

[50] Han B, Eskin E. Random-effects model aimed at discovering associations in meta-analysis of genome-wide association studies. The American Journal of Human Genetics. 2011;88(5):586–98. 10.1016/j.ajhg.2011.04.014 21565292 PMC3146723

[51] Locke AE, Kahali B, Berndt SI, Justice AE, Pers TH, Day FR, et al Genetic studies of body mass index yield new insights for obesity biology. Nature. 2015;518(7538):197–206. 10.1038/nature14177 25673413 PMC4382211

[52] Bandini LG, Schoeller DA, Cyr HN, Dietz WH. Validity of reported energy intake in obese and nonobese adolescents. The American journal of clinical nutrition. 1990;52(3):421–5. 10.1093/ajcn/52.3.421 2393004

[53] Yang Y, Mahaffey CL, Bérubé N, Nystuen A, Frankel WN. Functional characterization of fidgetin, an AAA-family protein mutated in fidget mice. Experimental cell research. 2005;304(1):50–8. 10.1016/j.yexcr.2004.11.014 15707573

[54] Cox GA, Mahaffey CL, Nystuen A, Letts VA, Frankel WN. The mouse fidgetin gene defines a new role for AAA family proteins in mammalian development. Nature genetics. 2000;26(2):198–202. 10.1038/79923 11017077

[55] Sanuki R, Omori Y, Koike C, Sato S, Furukawa T. Panky, a novel photoreceptor‐specific ankyrin repeat protein, is a transcriptional cofactor that suppresses CRX‐regulated photoreceptor genes. FEBS letters. 2010;584(4):753–8. 10.1016/j.febslet.2009.12.030 20026326

[56] Fox CS, Heard-Costa N, Cupples LA, Dupuis J, Vasan RS, Atwood LD. Genome-wide association to body mass index and waist circumference: the Framingham Heart Study 100K project. BMC medical genetics. 2007;8(1):S18. 17903300 10.1186/1471-2350-8-S1-S18 PMC1995618

[57] Kiel DP, Demissie S, Dupuis J, Lunetta KL, Murabito JM, Karasik D. Genome-wide association with bone mass and geometry in the Framingham Heart Study. BMC medical genetics. 2007;8(1):S14. 17903296 10.1186/1471-2350-8-S1-S14 PMC1995606

[58] Vasan RS, Larson MG, Aragam J, Wang TJ, Mitchell GF, Kathiresan S, et al Genome-wide association of echocardiographic dimensions, brachial artery endothelial function and treadmill exercise responses in the Framingham Heart Study. BMC medical genetics. 2007;8(1):S2. 17903301 10.1186/1471-2350-8-S1-S2 PMC1995617

[59] Fagerberg L, Hallström BM, Oksvold P, Kampf C, Djureinovic D, Odeberg J, et al Analysis of the human tissue-specific expression by genome-wide integration of transcriptomics and antibody-based proteomics. Molecular & Cellular Proteomics. 2014;13(2):397–406. 24309898 10.1074/mcp.M113.035600 PMC3916642

[60] Dina C, Meyre D, Gallina S, Durand E, Körner A, Jacobson P, et al Variation in FTO contributes to childhood obesity and severe adult obesity. Nature genetics. 2007;39(6):724–6. 10.1038/ng2048 17496892

[61] Speakman JR, Rance KA, Johnstone AM. Polymorphisms of the FTO gene are associated with variation in energy intake, but not energy expenditure. Obesity. 2008;16(8):1961–5. 10.1038/oby.2008.318 18551109

[62] Qi L, Kraft P, Hunter DJ, Hu FB. The common obesity variant near MC4R gene is associated with higher intakes of total energy and dietary fat, weight change and diabetes risk in women. Human molecular genetics. 2008;17(22):3502–8. 10.1093/hmg/ddn242 18697794 PMC2572696

[63] Chu AY, Workalemahu T, Paynter NP, Rose LM, Giulianini F, Tanaka T, et al Novel locus including FGF21 is associated with dietary macronutrient intake. Human molecular genetics. 2013;22(9):1895–902. 10.1093/hmg/ddt032 23372041 PMC3612009

[64] Spiegel T. Rate of intake, bites, and chews—the interpretation of lean–obese differences. Neuroscience & Biobehavioral Reviews. 2000;24(2):229–37. 10714386 10.1016/s0149-7634(99)00076-7

[65] Näslund E, Barkeling B, King N, Gutniak M, Blundell J, Holst J, et al Energy intake and appetite are suppressed by glucagon-like peptide-1 (GLP-1) in obese men. International journal of obesity. 1999;23(3):304–11. 10193877 10.1038/sj.ijo.0800818

[66] Druce MR, Small CJ, Bloom SR. Minireview: Gut peptides regulating satiety. Endocrinology. 2004;145(6):2660–5. 10.1210/en.2004-0089 15044353

[67] Ravussin E, Bogardus C. Energy balance and weight regulation: genetics versus environment. British Journal of Nutrition. 2000;83(S1):S17–S20. 10889787 10.1017/s0007114500000908

[68] Turnbaugh PJ, Hamady M, Yatsunenko T, Cantarel BL, Duncan A, Ley RE, et al A core gut microbiome in obese and lean twins. nature. 2009;457(7228):480–4. 10.1038/nature07540 19043404 PMC2677729

[69] Ley RE. Obesity and the human microbiome. Current opinion in gastroenterology. 2010;26(1):5–11. 10.1097/MOG.0b013e328333d751 19901833

[70] Friedman MI. Control of energy intake by energy metabolism. The American journal of clinical nutrition. 1995;62(5):1096S–100S. 7484927 10.1093/ajcn/62.5.1096S

[71] Freedman LS, Commins JM, Moler JE, Arab L, Baer DJ, Kipnis V, et al Pooled results from 5 validation studies of dietary self-report instruments using recovery biomarkers for energy and protein intake. American journal of epidemiology. 2014;180(2):172–88. 10.1093/aje/kwu116 24918187 PMC4082341

[72] Bedard D, Shatenstein B, Nadon S. Underreporting of energy intake from a self-administered food-frequency questionnaire completed by adults in Montreal. Public health nutrition. 2004;7(5):675–81. 10.1079/PHN2003578 15251058

[73] Ngui EM, Warner TD, Roberts LW. Perceptions of African-American health professionals and community members on the participation of children and pregnant women in genetic research. Public health genomics. 2014;17(1):23–32. 10.1159/000355359 24216722 PMC4014201

[74] Ledikwe JH, Rolls BJ, Smiciklas-Wright H, Mitchell DC, Ard JD, Champagne C, et al Reductions in dietary energy density are associated with weight loss in overweight and obese participants in the PREMIER trial. The American journal of clinical nutrition. 2007;85(5):1212–21. 10.1093/ajcn/85.5.1212 17490955

[75] Raben A, Agerholm-Larsen L, Flint A, Holst JJ, Astrup A. Meals with similar energy densities but rich in protein, fat, carbohydrate, or alcohol have different effects on energy expenditure and substrate metabolism but not on appetite and energy intake. The American journal of clinical nutrition. 2003;77(1):91–100. 10.1093/ajcn/77.1.91 12499328

