# Supplementary material for: A genome-wide association study of energy intake and expenditure
Source: PLoS One. 2018 Aug 2;13(8):e0201555. doi: 10.1371/journal.pone.0201555 (PMC6072034; doi:10.1371/journal.pone.0201555)
Supplement: S1 Table — (PDF) [file pone.0201555.s007.pdf]

S1 Table. Association between SNPs and daily energy balance among women, men, and meta-analyses combining women and men in the pooled GWAS.

Marker[a], alleles[b], chromosome[c], location[c], and genes[d]	Subset	Daily energy balance			
		EAF	Effect (95% CI)	P_{effect} value	P_{Het} value[e]
**Total population (*N*= 18,774)**					
**rs10876214** (T, C)	Female	0.34	5 (-6, 17)	0.37	
12q13 (52257245)	Male	**0.32**	**56 (37, 75)**	**1.22×10^{-8}**	
ANKRD33	Overall	0.33	19 (9, 29)	1.86×10^{-4}	1.12×10^{-5}
**Overweight and obese population (*N* = 10,583)**					
**rs2723689** (A, G)	Female	0.22	-7 (-26, 12)	0.49	
4q33 (169539246)	Male	**0.22**	**-78 (-107, -50)**	**8.02×10^{-8}**	
	Overall	0.22	-29 (-45, -13)	3.86×10^{-4}	4.44×10^{-5}

Note: Results from the unconditional logistic regression of the genotypes in the pooled GWAS for total subjects (12,031 women and 6,743 men. The analyses were adjusted for five principal components accounting for population substructure. Additionally, age, height, weight, and physical activity were adjusted for in energy intake. EAF, effect allele frequency; CI, confidence interval; Het, heterogeneity. [a]NCBI dbSNP identifier; [b]effect allele, reference allele; [c]chromosome and NCBI Human Genome Build 37 location; [d]closest genes, genes located within 25 kb; [e]Heterogeneity between women and men.
