# Supplementary material for: A genome-wide association study of energy intake and expenditure
Source: PLoS One. 2018 Aug 2;13(8):e0201555. doi: 10.1371/journal.pone.0201555 (PMC6072034; doi:10.1371/journal.pone.0201555)
Supplement: S2 Table — (PDF) [file pone.0201555.s008.pdf]

**S2 Table. Association between top SNPs that were identified from the total population and daily energy traits among overweight/obese and lean women, men, and meta-analyses combining women and men in the pooled GWAS.**

| Marker[a], alleles[b], chromosome[c], location[c], and genes[d] | Subset | Overweight and obese population ($N$ = 10,583) | | | | Lean population ($N$ = 10,583) | | | | |
|---|---|---|---|---|---|---|---|---|---|---|
| | | EAF | Effect (95% CI) | $P_{effect}$ value | $P_{Het}$ value[e] | EAF | Effect (95% CI) | $P_{effect}$ value | $P_{Het}$ value[e] | $P_{Het}$ value[f] |
| **Daily energy intake** | | | | | | | | | | |
| **rs10876214** (T, C) | Female | 0.34 | 7 (-10, 23) | 0.43 | | 0.34 | 4 (-13, 20) | 0.67 | | |
| 12q13 (52257245) | Male | 0.32 | 50 (25, 75) | $7.60 \times 10^{-5}$ | | 0.31 | 65 (36, 94) | $1.15 \times 10^{-5}$ | | |
| *ANKRD33* | Overall | 0.33 | 20 (6, 33) | 0.005 | 0.004 | 0.33 | 19 (4, 33) | 0.01 | 0.0003 | 0.92 |
| **rs9669605** (T, G) | Female | 0.32 | 8 (-9, 24) | 0.37 | | 0.31 | 4 (-13, 21) | 0.65 | | |
| 12q13 (52254674) | Male | 0.31 | 44 (19, 68) | $6.02 \times 10^{-4}$ | | 0.30 | 65 (36, 94) | $1.16 \times 10^{-5}$ | | |
| | Overall | 0.32 | 18 (5, 32) | 0.008 | 0.02 | 0.31 | 19 (4, 33) | 0.01 | 0.0003 | 0.95 |
| **rs10783478** (A, G) | Female | 0.29 | 1 (-16, 18) | 0.91 | | 0.28 | 3 (-14, 21) | 0.70 | | |
| 12q13 (52232476) | Male | 0.28 | 46 (21, 72) | $3.55 \times 10^{-4}$ | | 0.27 | 62 (32, 92) | $5.97 \times 10^{-5}$ | | |
| *FIGNL2* | Overall | 0.29 | 15 (1, 29) | 0.039 | 0.004 | 0.28 | 18 (3, 33) | 0.02 | 0.001 | 0.77 |
| **rs111431452** (A, T) | Female | 0.02 | 128 (67, 189) | $4.19 \times 10^{-5}$ | | 0.02 | -1 (-67, 66) | 0.98 | | |
| 1p31 (112056878) | Male | 0.02 | 189 (93, 286) | $1.27 \times 10^{-4}$ | | 0.02 | 9 (-109, 127) | 0.88 | | |
| *ADORA3* | Overall | **0.02** | **145 (94, 197)** | **$3.59 \times 10^{-8}$** | 0.29 | 0.02 | 2 (-56, 59) | 0.96 | 0.89 | 0.0003 |
| **Daily energy expenditure** | | | | | | | | | | |
| **rs142343672** (A, G) | Female | 0.01 | 265 (139, 390) | $3.50 \times 10^{-5}$ | | 0.01 | -74 (-168, 21) | 0.13 | | |
| 11p15 (17871273) | Male | 0.01 | -11 (-272, 250) | 0.93 | | 0.01 | -164 (-444, 116) | 0.25 | | |
| *LOC107984317* | Overall | 0.01 | 213 (100, 326) | $2.21 \times 10^{-4}$ | 0.06 | 0.01 | -83 (-172, 7) | 0.07 | 0.55 | |
| **rs146169233** (T, C) | Female | 0.01 | 80 (-19, 179) | 0.11 | | 0.01 | -38 (-121, 46) | 0.38 | | |
| 16p13 (9158320) | Male | 0.01 | -68 (-246, 110) | | | | | | | |
| | Overall | 0.01 | 45 (-42, 131) | 0.31 | 0.15 | 0.01 | -38 (-121, 46) | 0.38 | 1.00 | |
| **rs61957289** (C, T) | Female | 0.01 | 134 (39, 229) | 0.006 | | 0.01 | 24 (-60, 109) | 0.58 | | |
| 13q22 (74400573) | Male | 0.01 | -54 (-250, 142) | 0.59 | | 0.01 | -156 (-463, 152) | 0.32 | | |
| *KLF12* | Overall | 0.01 | 98 (12, 183) | 0.02 | 0.09 | 0.01 | 112 (-70, 93) | 0.78 | 0.27 | |
| **rs62131523** (G, A) | Female | 0.96 | -14 (-36, 8) | 0.21 | | 0.04 | 4 (-15, 22) | 0.69 | | |
| 2p24 (17746338) | Male | **0.96** | **124 (80, 169)** | **$3.41 \times 10^{-8}$** | | 0.04 | -29 (-78, 19) | 0.24 | | |
| *VSNL1* | Overall | 0.96 | 13 (-6, 33) | 0.19 | $3.71 \times 10^{-8}$ | 0.04 | -0.4 (-18, 17) | 0.96 | 0.21 | |
| **rs7162556** (A, G) | Female | 0.10 | 2 (-11, 16) | 0.73 | | 0.09 | -4 (-16, 7) | 0.47 | | |

| | | | | | | | | | |
|---|---|---|---|---|---|---|---|---|---|
| 15q25 (80675244) | Male | **0.10** | **-71 (-97, -46)** | **$3.66 \times 10^{-8}$** | | 0.10 | -25 (-52, 2) | 0.07 | |
| | Overall | 0.10 | -14 (-25, -2) | 0.03 | $4.45 \times 10^{-7}$ | 0.09 | -8 (-18, 3) | 0.16 | 0.16 |

*Note:* Results from the unconditional logistic regression of the genotypes in the pooled GWAS for total subjects (12,031 women and 6,743 men) and overweight/obese subjects only (6,563 women and 4,020 men). The analyses were adjusted for five principal components accounting for population substructure. Additionally, age, height, weight, and physical activity were adjusted for in energy intake. EAF, effect allele frequency; CI, confidence interval; Het, heterogeneity. [a]NCBI dbSNP identifier; [b]effect allele, reference allele; [c]chromosome and NCBI Human Genome Build 37 location; [d]closest genes, genes located within 25 kb; [e]Heterogeneity between women and men; [f]Heterogeneity between overweight/obese and lean subjects.
