# Supplementary material for: A genome-wide association study of energy intake and expenditure
Source: PLoS One. 2018 Aug 2;13(8):e0201555. doi: 10.1371/journal.pone.0201555 (PMC6072034; doi:10.1371/journal.pone.0201555)
Supplement: S3 Table — (PDF) [file pone.0201555.s009.pdf]

S3 Table. Information of ßs and standard errors of SNP-set analysis for the association between body mass index and energy traits in total population.

Marker Name	Effect Allele	Ref Allele	Beta$_{BMI}$	Beta$_{Energy\ Intake}$	S.E.$_{Energy\ Intake}$	Beta$_{Energy\ Expenditure}$	S.E.$_{Energy\ Expenditure}$
19:45395619	A	G	0.026	0.671	1.147	5.480	7.488
1:177889480	G	A	0.048	0.689	0.890	-2.876	5.827
1:201784287	C	A	0.020	0.085	0.710	7.993	4.633
15:68077168	T	C	0.031	-0.101	0.833	-3.939	5.431
4:145659064	T	C	0.036	1.042	1.326	12.720	8.680
17:5283252	G	A	0.019	-0.600	0.766	-4.168	5.004
10:114758349	C	T	0.023	1.567	0.776	1.581	5.068
4:103188709	T	C	0.048	1.221	1.409	-13.274	9.176
19:46202172	C	T	0.036	0.362	1.017	3.932	6.640
14:25928179	C	A	0.023	1.083	0.780	-2.435	5.093
6:40348653	G	A	0.019	-0.791	0.786	6.673	5.140
16:19935389	G	A	0.040	-1.291	1.015	13.039	6.663
2:143043285	T	C	0.025	1.451	1.061	1.348	6.924
16:31129895	A	G	0.019	0.882	0.730	-1.736	4.770
9:129460914	A	G	0.017	-0.680	0.704	2.095	4.605
1:49589847	A	G	0.023	0.246	0.741	-6.900	4.844
1:110154688	C	T	0.066	0.028	2.087	2.374	13.695
19:18454825	A	G	0.019	1.778	0.799	-5.862	5.225
8:76806584	T	C	0.022	-0.376	0.764	-10.410	4.994
10:104869038	C	T	0.031	-0.592	1.229	2.284	8.057
2:181550962	T	C	0.018	1.160	0.727	-3.712	4.743
11:47650993	T	C	0.026	-0.923	0.719	-6.089	4.693
13:28017782	T	C	0.030	-2.042	0.920	0.850	6.026
14:29736838	C	A	0.021	-0.860	0.754	1.792	4.944
6:50845490	G	A	0.045	-0.972	0.938	-6.329	6.131
9:15634326	T	C	0.018	1.021	0.706	-8.515	4.620
1:75002193	G	A	0.024	-0.681	0.708	2.888	4.642
2:59305625	T	C	0.023	-0.652	0.775	1.233	5.059
3:61236462	C	T	0.020	-0.035	0.719	-3.847	4.708

16:3627358	T	C	0.023	1.241	0.844	3.481	5.518
14:30515112	T	C	0.049	0.068	1.739	-11.588	11.435
3:141275436	T	G	0.048	1.694	1.494	2.054	9.710
12:122781897	G	A	0.031	0.441	1.335	-0.490	8.890
3:85807590	G	T	0.030	-2.225	0.870	3.689	5.674
6:163033350	A	G	0.028	-0.062	1.087	4.184	7.126
9:111932342	C	T	0.017	1.279	0.742	-1.081	4.870
1:96924097	T	C	0.022	-0.561	0.719	-0.264	4.692
6:108977663	C	T	0.019	-0.483	0.765	-9.917	5.001
11:27684517	A	G	0.041	-0.762	0.864	10.660	5.620
18:21104888	C	T	0.017	-0.266	0.716	5.909	4.671
3:25106437	G	A	0.019	-0.273	0.717	-1.888	4.689
18:56883319	T	G	0.022	-0.992	0.943	-3.233	6.129
1:50559820	C	T	0.018	0.191	0.722	-11.414	4.715
1:78446761	A	G	0.021	0.169	0.750	-0.509	4.918
18:57829135	C	T	0.056	-1.659	0.828	1.421	5.431
5:75015242	T	G	0.026	-0.071	0.744	3.792	4.856
10:87410904	G	A	0.040	-1.952	1.657	-0.128	10.800
16:28889486	A	C	0.031	2.079	0.752	-7.203	4.949
4:45182527	G	A	0.040	-0.843	0.713	-7.354	4.662
17:78615571	G	A	0.018	-1.316	0.709	-6.493	4.634
6:34563164	G	A	0.022	-0.015	0.787	-3.167	5.135
2:26928811	A	G	0.021	-0.147	0.797	10.063	5.217
2:25150296	G	A	0.031	-0.315	0.703	-4.751	4.602
2:632348	G	A	0.060	-0.039	0.910	0.184	5.920
8:85079709	C	T	0.019	-2.201	0.840	-4.301	5.507
16:53803574	A	T	0.082	-0.687	0.714	2.243	4.672
19:34309532	G	A	0.018	-0.664	0.757	-4.092	4.946
3:81792112	A	C	0.019	-0.433	0.753	6.733	4.940
3:185824004	C	T	0.045	0.282	1.045	-18.007	6.828
10:102395440	C	T	0.025	0.858	0.870	-4.656	5.683
9:120378483	T	C	0.019	-0.147	0.708	1.617	4.632
12:50247468	A	G	0.032	0.199	0.725	-0.121	4.735

11:43864278	T	C	0.020	1.330	0.815	-8.423	5.327
15:51748610	A	G	0.018	0.214	0.714	0.204	4.674
19:47569003	A	G	0.028	-0.409	0.849	2.283	5.546
1:72751185	C	T	0.033	-0.439	0.735	-11.950	4.813
2:213413231	G	A	0.022	-0.671	0.792	-6.539	5.191
14:79899454	T	C	0.024	-0.939	0.711	0.720	4.627
13:54102206	A	G	0.033	-0.550	1.046	-9.171	6.832
11:8673939	G	C	0.021	-0.879	0.731	-5.497	4.775
16:28333411	A	G	0.021	2.561	1.108	0.013	7.230
7:75163169	G	A	0.020	0.690	0.734	-5.742	4.793
2:63053048	G	A	0.017	0.094	0.715	0.281	4.673
11:115022404	G	A	0.022	2.308	0.704	0.110	4.598
4:77129568	G	C	0.031	-1.712	0.970	-4.047	6.324
9:28414339	G	A	0.025	-1.178	0.764	1.444	4.992

Notes: BMI-related SNPs were identified from Giant study. The corresponding ßs and standard errors of energy intake and energy expenditure were extracted from our study. S.E., standard error. Re allele, reference allele.
